# Asparaginyl Endopeptidase (Legumain) Supports Human Th1 Induction via Cathepsin L-Mediated Intracellular C3 Activation

**DOI:** 10.3389/fimmu.2018.02449

**Published:** 2018-10-24

**Authors:** Simon Freeley, John Cardone, Sira C. Günther, Erin E. West, Thomas Reinheckel, Colin Watts, Claudia Kemper, Martin V. Kolev

**Affiliations:** ^1^School of Immunology and Microbial Sciences, King's College London, London, United Kingdom; ^2^Institut für Medizinische Virologie, University of Zurich, Zurich, Switzerland; ^3^Laboratory of Molecular Immunology and Immunology Center, National Heart, Lung and Blood Institute, Bethesda, MD, United States; ^4^Faculty of Medicine, Institute of Molecular Medicine and Cell Research, Albert-Ludwigs University Freiburg, and German Cancer Consortium (DKTK), Freiburg, Germany; ^5^Division of Cell Signaling & Immunology, School of Life Sciences, University of Dundee, Dundee, United Kingdom; ^6^Institute for Systemic Inflammation Research, University of Lübeck, Lübeck, Germany

**Keywords:** complement, CD46, T cell, cathepsin L, AEP, legumain

## Abstract

Autocrine activation of the complement receptors C3aR and CD46 by complement activation components C3a and C3b produced through C3 cleavage by the protease cathepsin L (CTSL) during T cell stimulation is a requirement for IFN-γ production and Th1 induction in human CD4^+^ T cells. Thus, lack of autocrine CD46 activation, such as in CD46-deficient patients, is associated with defective Th1 responses and recurrent infections. We have identified *LGMN* [the gene coding for legumain, also known as asparaginyl endopeptidase (AEP)] as one of the key genes induced by CD46 co-stimulation during human CD4^+^ T cell activation. AEP processes and activates a range of proteins, among those α1-thymosin and CTSL, which both drive intrinsically Th1 activity—but has so far not been described to be functionally active in human T cells. Here we found that pharmacological inhibition of AEP during activation of human CD4^+^ T cells reduced CTSL activation and the CTSL-mediated generation of intracellular C3a. This translated into a specific reduction of IFN-γ production without affecting cell proliferation or survival. In line with these findings, CD4^+^ T cells isolated from *Lgmn*^−/−^ mice also displayed a specific defect in IFN-γ secretion and Th1 induction. Furthermore, we did not observe a role for AEP-driven autocrine α1-thymosin activation in T cell-derived IFN-γ production. These data suggest that AEP is an “upstream” activator of the CTSL-C3-IFN-γ axis in human CD4^+^ T cells and hence an important supporter of human Th1 induction.

## Introduction

Liver-derived, serum-circulating complement is a critical part of innate immunity mediating protection against invading pathogens. Proteolytic activation of the complement key components C3 into C3a and C3b and C5 into C5a and C5b upon pathogen sensing in blood leads to opsonization and removal of invading microbes, mobilization of innate immune cells, and induction of the general inflammatory response (1). However, it is now also acknowledged that complement serves an equally central role in the direct regulation of human CD4^+^ T cell responses: signals delivered by the human-specific C3b-binding complement regulator/receptor CD46 (membrane cofactor protein, MCP) and the C3a receptor (C3aR) are essential for the induction of IFN-γ in human CD4^+^ T cells (2, 3). Furthermore, together with IL-2, CD46 also mediates IL-10 co-expression in expanded Th1 cells and, via this, the switch toward a (self) regulatory contraction phase (4) (Figure S1A). Unexpectedly, CD46 and C3aR-mediated activation of T cells is independent of liver-derived C3, but driven in an autocrine fashion by the C3 activation fragments C3b and C3a generated through cathepsin L (CTSL)-mediated cleavage of intracellular C3 that shunts to the cell surface upon T cell receptor (TCR) activation (4, 5). Thus, lack of autocrine CD46 activation, such as in CD46-deficient patients, results in reduced Th1 responses and recurrent infections (2), whilst uncontrolled autocrine C3 activation and dysregulated CD46 engagement contributes to hyperactive Th1 responses in autoimmunity (4–6).

Importantly, as increased intracellular CTSL-driven C3 activation can be pharmacologically targeted by a cell-permeable CTSL inhibitor leading to normalization of hyper-Th1 activity at least *in vitro* (5), we aimed at better understanding the modes of CTSL activation in T cells. When analyzing gene arrays derived from resting or TCR andCD46 activated human CD4^+^ T cells (7), we noted that asparaginyl endopeptidase (AEP or legumain) was strongly expressed in T cells and further augmented upon CD46 co-stimulation. AEP is an asparagine-specific cysteine protease found in lysosomes and plays an important but non-exclusive role in the first step of invariant chain of major histocompatibility class II (MHC II) processing in antigen presenting cells (APC) (8). AEP also processes and activates a range of additional proteins. Among those are α1-thymosin and CTSL, which both drive intrinsically Th1 activity (5, 9), and AEP-deficient mice accordingly exhibit a defect in the maturation of catepsins B, H, and L in kidney cells (10). However, so far, AEP activity has not been described in human T cells.

Here we describe for the first time a role for AEP in human CD4^+^ T cells and its specific requirement for normal Th1 induction.

## Materials and methods

### Healthy donors

Blood samples were obtained with ethical approvals at King's College London (Wandsworth Research Ethics Committee, REC# 09/H0803/154). CD4^+^ T cells were purified from buffy coats (NHSBT, Tooting, UK) or blood samples from healthy volunteers after informed consent.

### Mice

Wild type and *Lgmn*^−/−^mice were generated by Drs. Thomas Reinheckel and Colin Watts as previously described (11). All animals were maintained in accredited BSL2 facilities at KCL and experiments performed in compliance with animal study proposals approved by KCL.

### Antibodies, proteins, and inhibitors

For a list of antibodies, proteins and inhibitors utilized, see Supplementary Materials.

### T cell isolation and activation

For a protocol of human and mouse CD4^+^ T cell isolation and activation, see Supplementary Materials.

### Cytokine measurements

Cytokine secretion by human and mouse T cells was measured using the human and mouse Th1/Th2/Th17 Cytokine Bead Arrays (BD Biosciences, Oxford, UK) or the combined Secretion Assays for human IFN-γ and IL-10 purchased from Miltenyi Biotec (Bergisch Gladbach, Germany) according to the manufacturers' provided protocols.

### Confocal microscopy

For a detailed protocol on the confocal microscopy performed here including the measurement of the Pearson's Correlation Coefficient, refer to Supplementary Materials.

### Western blotting

For a detailed protocol on the Western botting procedures and analyses utilized, see Supplementary Materials.

### Statistical analyses

Statistical analyses were performed on GraphPad Prism 7 (La Jolla, CA). Data are presented as mean ± SEM and compared using paired t-tests or one-way ANOVA with a Tukey multiple comparison *post-hoc* test, as appropriate. p < 0.05 denoted statistical significance throughout.

## Results

### AEP is required for normal Th1 induction in human and mouse CD4^+^ T cells

**Figure 1:**
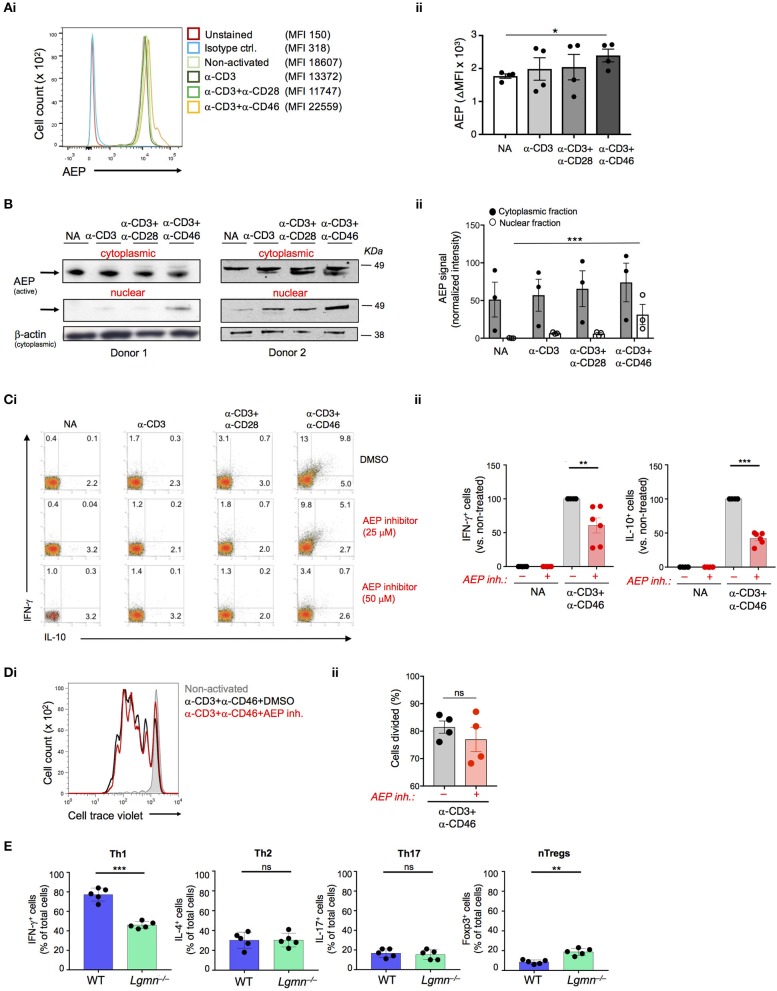
AEP is required for normal IFN-γ production in human and mouse CD4^+^ T cells. **(A,B)** CD46 drives AEP expression and nuclear translocation. Human CD4^+^ T cells were left non-activated (NA) or activated with the depicted antibody combinations and AEP expression assessed 36 h post activation by **(Ai)** FACS with **(Aii)** statistical analyses and **(Bi)** Western blotting of the cytoplasmic and nuclear fractions with **(Bii)** respective statistical analyses of the signals by densitometry. Shown are one representative FACS and two Western blot experiments of *n* = 3 using a different donor each time. **(C)** AEP inhibition suppresses human Th1 induction. T cells were activated as described under “A” with or without 25 or 50 μM of a specific AEP inhibitor and IFN-γ and IL-10 (co)secretion measured 36 h post activation. **(Ci)** shows FACS data derived from a representative donor whilst **(Cii)** summarizes the analyses for the shown activation conditions of *n* = 6 donors. **(D)** AEP inhibition does not affect cell proliferation. Cell trace violet-labeled CD4^+^ T cells were CD3+CD46-activated in the presence or absence of 50 μM AEP inhibitor and cell proliferation measured at 6 d post activation. **(Di)** Shows a representative FACS profile and **(Dii)** the accompanying statistical analysis from four different experiments (*n* = 4). **(E)** AEP is also required for normal Th1 induction in mice. Naïve CD4^+^ T cells isolated from wild type (WT) or AEP-deficient (*Lgmn*^−/−^) mice (*n* = 5) were activated for 6 days under Th1, Th2, or Th17 skewing conditions and the total numbers of IFN-γ (Th1), IL-4 (Th2), or IL-17-positive (Th17) cells assessed by intracellular cytokine staining. The number of FoxP3-positive natural regulatory T cells (nTregs) was assessed in activated cell cultures without addition of skewing cytokines/antibodies. Error bar graphs represent mean ± SEM. ^*^P < 0.05, ^**^P < 0.01, ^***^P < 0.001; ns, not significant.

Gene expression analyses performed on resting and CD3+CD46-activated human CD4^+^ T cells suggested the expression modulation of the *LGMN* gene, encoding the endopeptidase AEP (7). Indeed, resting CD4^+^ T cells contained high levels of AEP protein in the cytoplasm and CD46-mediated co-stimulation during TCR activation further increased AEP protein levels but simultaneously induced the nuclear translocation of a proportion of AEP (Figures 1A,B). CD3+CD46-activation of T cells is a strong and specific inducer of human Th1 responses (2). The addition of increasing doses of a specific AEP inhibitor (12) during CD3+CD46 activation significantly reduced the percentage of actively IFN-γ-secreting cells as well as their switching into the IL-10-producing contracting phase in cultures in a dose-dependent manner (Figure 1C and Figure S1B). The observed reduction of IFN-γ and IL-10 secretion also in CD3 and CD3+CD28-activated T cells upon AEP inhibition was expected, as TCR stimulation and CD28-costimulation function upstream of CD46 and trigger increased intracellular CTSL-mediated C3b generation and “background” CD46 engagement (5). Of note, neither cell proliferation, viability nor production of Th2 cytokines such as IL-4 were affected by AEP inhibition and Th17 responses were only reduced significantly under the CD3+CD46 stimulation condition (Figure 1D and Figures S1B,C).

We confirmed a role for AEP in the production and secretion of IFN-γ by CD4^+^ T cells also in mice. Importantly, mice do not express CD46 on somatic tissue (13) and a functional homologue that drives Th1 induction—as shown for CD46 in human T cells—has so far not been identified in mouse T cells (14). Thus, purified CD4^+^ T cells from either wild type (WT) or AEP-deficient (*Lgmn*^−/−^) were activated under Th1, Th2, or Th17-skewing conditions and the percentage of IFN-γ, IL-4, and IL-17-positive cells, as well as secretion of these cytokines into culture media assessed. AEP deficiency was accompanied by a specific and significant reduction in Th1 induction (Figure 1E). We also observed an increase in the circulating natural FoxP3^+^ regulatory T cell (Treg) pool in *Lgmn*^−/−^ animals (Figure 1E and Figure S1D).

These data indicate that AEP activity is required for normal Th1 induction in mice and men.

### AEP is required for CTSL-mediated C3 activation in human CD4^+^ T cells

**Figure 2:**
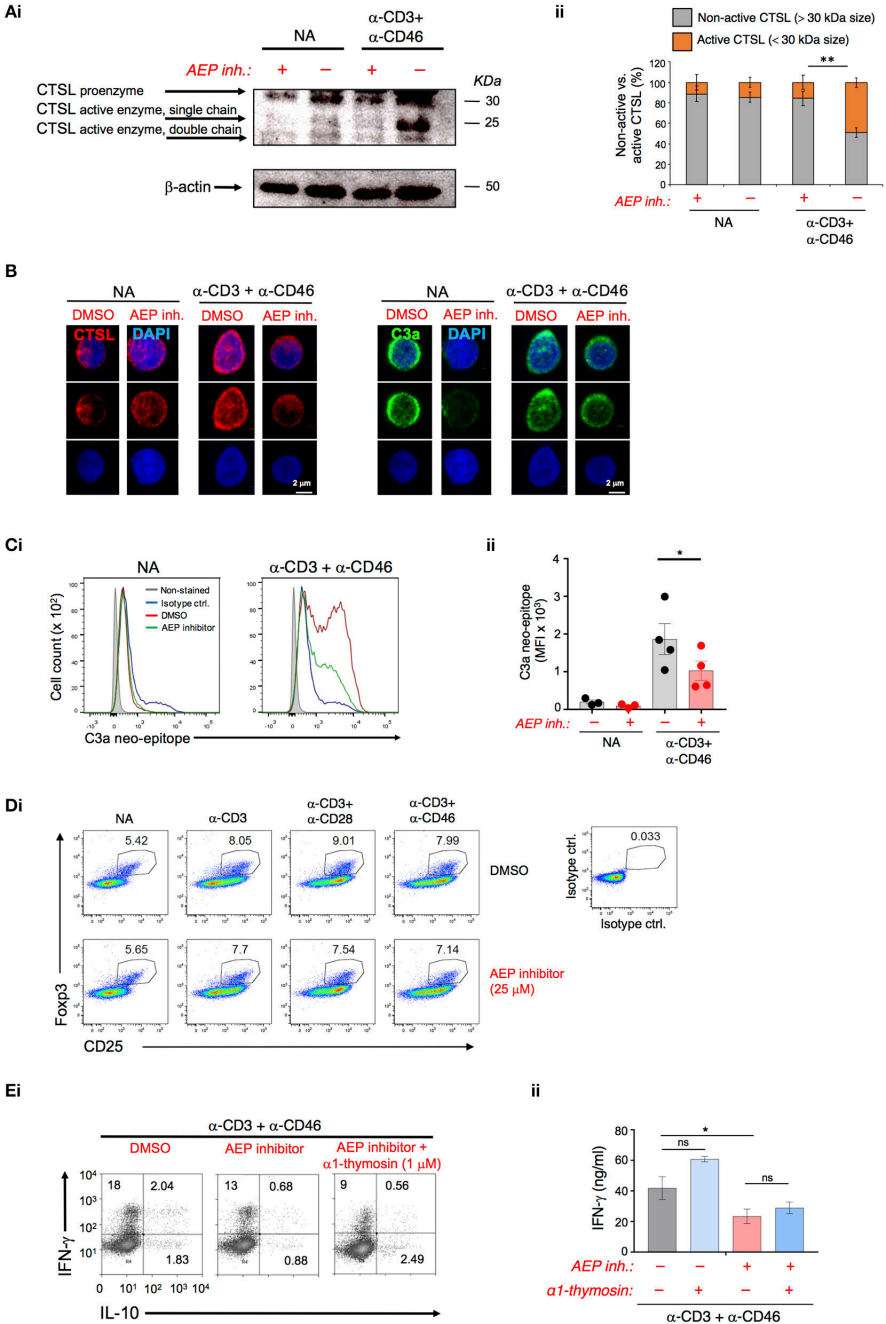
AEP activity drives cathepsin L-mediated C3 activation in human CD4^+^ T cells. **(A–C)** AEP inhibition reduces the generation of active cathepsin L (CTSL) and CTSL-mediated C3a production. T cells were left resting (NA) or CD3+CD46-activated with or without 50 μM AEP inhibitor for 36 h. Appearance of the CTSL active single or double chains generated from cleavage of the proenzyme form was measured by **(Ai)** Western blotting with **(Aii)** appropriate densitometric analyses of the percentage of active (<30 kDa size bands) vs. non-active (>30 kDa size bands) CTSL forms. **(B)** confocal microscopy (left panels). The generation of CTSL-dependent intracellular C3a was measured by **(B)** confocal microscopy (right panels) and **(Ci)** FACS with subsequent **(Cii)** statistical analysis. Data shown in “A–C” are derived from *n* = 4 healthy donors. **(D)** T cells were activated under the depicted conditions and Foxp3 protein expression measured at 36 h post activation via FACS analysis. Shown is one representative experiment of two similarly performed. **(E)** Autocrine AEP-mediated α1-thymosin activation is not required for Th1 induction. T cells were activated as under “A” with the addition of 1 μM α1-thymosin as indicated and **(Di)** IFN-γ and IL-10-producing cells enumerated and **(Dii)** IFN-γ secretion measured 36 h post activation (*n* = 3). Error bar graphs represent mean ± SEM. ^*^P < 0.05, ^**^P < 0.01; ns, not significant.

In human CD4^+^ T cells, CTSL is key in activating C3 and thereby providing the ligands for autocrine CD46 and C3aR activation needed for Th1 induction (5) (Figure S2A). CTSL is usually synthesized as inactive pre-proenzyme and requires proteolytic activation (first removal of the pre-peptide and then processing into active single or double-chain forms) by other proteases (Figure S2B) (15). AEP has been shown to be involved in the processing of single-chain CTSL into the double-chain form in mice (10, 16). Inhibition of AEP slightly in resting cells but significantly during CD3+CD46 activation reduced the generation of active single and double chain CTSL forms as assessed by Western blotting and confocal microscopy in human T cells (Figures 2A,B). Importantly, the decrease in the generation of the CTSL active forms by AEP inhibition led also to a proportional and significant reduction in the activation of C3 as measured by the appearance of the C3a neo-epitope in CD3+CD46-stimulated T cells (Figures 2B,C and Figure S2C). A recent study found that AEP cleaves FoxP3 in mouse induced regulatory T cells and confirmed increased Tregs in *Lgmn*^−/−^ mice (17). We did, however, not observe changes in Foxp3 levels in resting or activated human CD4^+^ T cells upon AEP inhibition (Figure 2D).

AEP also processes pro-thymosin α into the active α1-thymosin form (18) and α1-thymosin has been implicated in human CD4^+^ T cell activation and modulation of their cytokine production (19). However, the addition of recombinant α1-thymosin during T cell activation in the presence of the AEP inhibitor failed to rescue defective Th1 induction (Figure 2E). Human CD4^+^ T cells upregulate major histocompatibility class II (MHC II) molecules (and their maturation require CTSL cleavage) upon activation (20)—however, this process also remained unaffected by AEP inhibition (Figure S2D).

Together these data suggest that AEP activity in human CD4^+^ T cells drives Th1 induction via proteolytic activation of CTSL, which in turn increases C3 activation and autocrine CD46 and C3aR activation.

## Discussion

CTSL-mediated activation of intracellular C3 into C3a and C3b—which then drive the autocrine engagement of the C3aR and CD46, respectively—is critical to normal Th1 cell responses. CD46 deficiency is accompanied hence by recurrent infections throughout life and patients with serum C3 deficiency have recurrent infections early in life. Of note, whilst C3 deficiency was long thought to be based solely on reduced pathogen lytic activity and impairment of innate immunity, it is now understood that C3-deficiency indeed also impairs adaptive immunity (21). Increased CTSL-mediated C3 activation, on the other hand, drives Th1 hyper-activity in rheumatoid arthritis and in systemic lupus erythematosus (5, 22). Pharmacological inhibition of intracellular CTSL activity can normalize dysregulated Th1 responses, demonstrating that this pathway is therapeutically amendable. Thus, understanding how CTSL activity is regulated adds to our better understanding of Th1 biology and may also provide additional needed therapeutic targets to control aberrant Th1 immunity.

Here we identified AEP as an enzyme that processes CTSL into its active forms in human CD4^+^ T cells. Inhibition of AEP activity reduced the appearance of the CTSL active forms, the generation of C3a in T cells and the induction of IFN-γ-secreting cells by about 50%. This strongly suggests that AEP functions upstream of the CTSL-C3 axis and is an integral part of human Th1 initiation. AEP processes only the CTSL single chain into the double chain form in mouse kidney cells (10). It is currently unclear whether CTSL single chain-generating activity of AEP is human (T cell) specific and/or if there is a difference in the CTSL single vs. double chain forms' ability or potency to cleave C3. The latter is feasible since single chain and double chain CTSL can indeed have distinct substrate specificities (23).

This is the first description of a role for AEP in human Th1 biology. A previous study identified a role for AEP, together with the protease inhibitor SerpinB1, in the regulation of CTSL in mouse CD4^+^ T cells (24). Interestingly though, AEP blockage with an inhibitor (the authors did not utilize *Lgmn*^−/−^ animals) in presence of *Serpinb1* deficiency negatively controlled CTSL-mediated Th17 induction without affecting Th1 responses. The difference in outcome may be due to additional, not yet defined, functions of SerpinB in Th17 induction. Here, using *Lgmn*^−/−^ animals, we found that, similar to human CD4^+^ T cells, mouse Th1 responses are reduced while Th17 responses remain unaffected. Of note, although mouse Th1 induction also requires intracellular C3 activation, CD4^+^ T cells from *Ctsl*^−/−^ mice produce normal IFN-γ levels (5), indicating that C3 activation in mice can occur in a CTSL-independent fashion. In line with this, C3a levels are unchanged in *Lgmn*^−/−^ animals (Figure S2E). Given these differences, it seems unlikely that AEP regulates mouse Th1 induction via a similar CTSL-C3 axis.

Previous work also noted increased natural and induced Tregs in *Lgmn*^−/−^ mice in line with the observation that AEP stabilizes regulatory T cells through nuclear Foxp3 cleavage (17, 25). Although CD46-activation indeed induced the nuclear translocation of both AEP and CTSL (Figure 1B and Figure S2F) we did not observe an effect of AEP inhibition on Foxp3—or other confirmed AEP/CTSL targets such as α1-thymosin or MHC II—in human CD4^+^ T cells. It is possible that the AEP inhibitor used here only affects certain AEP activities. Patients with genetic AEP deficiency that could be of help to better define AEP's roles in humans have so far not been described and we would therefore not exclude that AEP (and CTSL) serves additional functions aside from C3 activation in supporting Th1 activation.

In sum, AEP activity is important to normal Th1 induction in mice and men—the exact modes of action of AEP in these cells, however, seem to be species-specific and require further investigation.

## Author contributions

TR, CW, MVK, and CK conceived the study, helped designing key experiments, discussed the data, and drafted the manuscript. SF, JC, EEW, SCG, and MVK designed and performed experiments, analyzed and interpreted the data, and finalized the manuscript. CK and MVK share the last authorship. All authors approved the final version of the manuscript and agreed to be accountable to all aspects of this work.

### Conflict of interest statement

The authors declare that the research was conducted in the absence of any commercial or financial relationships that could be construed as a potential conflict of interest.
