# Supplementary material for: Asparaginyl Endopeptidase (Legumain) Supports Human Th1 Induction via Cathepsin L-Mediated Intracellular C3 Activation
Source: Front Immunol. 2018 Oct 24;9:2449. doi: 10.3389/fimmu.2018.02449 (PMC6207624; doi:10.3389/fimmu.2018.02449)
Supplement: Supplementary file 1 [file Data_Sheet_1.docx]

**Asparaginyl endopeptidase (legumain) supports**

**human Th1 induction via**

**cathepsin L-mediated intracellular C3 activation**

Simon Freeley, John Cardone, Sira Carolin Günther, Erin E. West, Thomas Reinheckel, Colin Watts, Claudia Kemper, and Martin Kolev

**Supplementary Material:**

1. **Detailed Materials and Methods**
2. **Supplementary Figure 1**
3. **Supplementary Figure 2**
4. **Supplemental references**

**Detailed Materials and Methods**

### Antibodies, proteins and inhibitors

Cell-stimulating monoclonal antibodies to human CD4^+^ T cells were bought from BD Biosciences, San Diego, CA (anti-hCD28, CD28.2), purified from a specific hybridoma (anti-hCD3; OKT-3) or generated in-house (anti-CD46; TRA-2-10) (Wang et al., 2000). Mouse T cells were activated with anti-CD3 (145-2C11) and anti-CD28 (37.51) from Bio X Cell (West Lebanon, NH). The monoclonal antibody to human C3a/C3adesArg (clone 2991, ab11873) was purchased from Abcam (Cambridge, UK) and the antibody detecting the mouse C3a neo-epitope from Hycult Biotech (clone 3/11; Uden, The Netherlands). The AEP inhibitor was generated by Dr. Colin Watts as previously described (Loak et al., 2003) and utilized at 5, 25 or 50 μM concentrations as indicated. The anti-AEP antibody (ab232870) as well as the cathepsin L antibody (ab95154) were bought from Abcam. The anti-IFN-γ (502515 human and 505813 mouse, respectively) and anti-IL-10 antibodies (506804 human and 505010 mouse) were obtained from Biolegend (San Diego, CA). Recombinant α1-thymosin protein was purchased from from Iris Biothech GmbH (Markytdwitz, Germany). Recombinant mouse IL-12 (577008), IL-4 (574308), and IL-1β (575108) as well as the and anti-IFN-γ (575308) and anti-IL-4 (504108) antibodies used for mouse Th1, Th2 and Th17 induction were obtained from Biolegend (San Diego, CA). Recombinant TGF-β (P0402) was purchased from Biotechne (Abingdon, UK) and the anti-mouse Foxp3 antibody was purchased from BD Biosciences (56310; Wokingham, UK). Secondary antibodies used in Western blotting were from BioRad: donkey anti-rabbit IgG HRP (644005) or goat anti-mouse IgG HRP (0300-0108P). The anti-human Foxp3-FITC antibody (clone #PCH101) was from eBioscience (Waltham, MA), the HLA-DR PerpCy5.5 (clone # L243) and anti-CD4-PAC blue (clone #OKT4) antibodies from Biolegend, the CD25-PE (clone # 2A3) antibody from BD Biosciences (Franklin Lakes, NJ), whilst the Live/dead near IR (#L10119) and the Cell trace violet  (# C34557) staining reagents were both purchased from Invitrogen (Waltham, MA).

**T cell isolation and activation**

*Human cells*: CD4^+^ T cells were isolated from blood using the MACS Human CD4^+^ Positive T cell Isolation Kit (Miltenyi, Biotech Ltd, Bisley, UK). Purity of bead-isolated T lymphocyte fractions was typically > 98%. T cells were activated for 36 h in 48-well (Greiner, Monroe, NC, reference 677180) culture plates at 2.5 – 3.0 x 10^5^ cells/well concentration in presence of 25 U/ml rhIL-2 in wells that had been coated with mAbs to CD3, CD3+CD28 or CD3+CD46 (2.0 μg/ml each). Cell viability was monitored using the [Promega™ CellTiter-Glo™ Luminescent Cell Viability Assay Kit](https://www.fishersci.ca/shop/products/promega-celltiter-glo-luminescent-cell-viability-assay-kit-6/p-107437) (Promega, Madison, WI) according to the manufacturer’s protocol.

*Mouse cells*: Single cell suspensions of spleen cells were generated and red blood cells lysed using ACK lysis buffer (Life Technologies, Carlsbad, CA). CD4^+^ T cells were isolated by negative selection using the Stem Cell Technologies EasySep^TM^ Mouse CD4^+^ T Cell Isolation Kit (Tukwila, WA). To separate naïve versus memory CD4^+^ T cells cells, T cells were sort-separated based on CD4^+^ CD44^+^ (memory) and CD4^+^ CD44^-^ (naïve) stainings. For *in vitro* T cell activation, 48-well plates were coated with 2 ug/ml anti-CD3 overnight at 4°C and CD4^+^ T cells (0.5 – 1.0 x 10^6^ per well of 48-well plates) were added to the appropriate wells. One ug/ml of anti-CD28 was added to the media to provide co-stimulation. For Th1, Th2 and Th17 induction, 20 ng/ml rIL-12, and 50 ng/ml anti-IL-4 (Th1), 40 ng/ml IL-4 and 50 ng/ml anti-IFN-γ (Th2) or 50 ng/ml anti-IL-4, 50 ng/ml anti-IFN-γ, 40 ng/ml TFG-β and 20 ng/ml IL-1β (Th17) were added. Cells were activated for 3 days and then media was replaced with fresh media only containing neutralizing antibodies for respective skewing conditions. Supernatants were taken at day 5 and 50 ng/ml PMA, 1μg/ml Ionomycin and 1x GolgiStop (BD) were added for 4 hours prior to staining and FACS analysis of intracellular IFN-γ, IL-4 and IL-17 expression.

**Cell proliferation assay**

Purified CD4^+^ T cells were labeled with 2 uM of Cell trace violet and then cultured *in vitro* either alone or with CD3 + CD46 stimulation for 6 days. On Day 6, cells were stained for CD4 and viability (live/dead near IR stain) and dilution of Cell trace violet on live cells was measured using a BD FACS CANTO II.

### Confocal microscopy

### Confocal microscopy staining and imaging was done as previously described using (Liszewski et al., 2013). Briefly, cells were fixed and permeabilized and then stained with appropriate primary antibodies over night at 4 ^o^C. Where indicated, subsequent staining with secondary antibodies was performed for 2 h at room temperature. Cells were then mounted with ProLong Gold (ThermoFisher, Paysley, UK) and images obtained using the KCL Nikon Imaging Centre by confocal fluorescence microscopy using an A1R SI Confocal Microscope with a x60 objective (Nikon^TM^, Surrey, UK). For the determination of the Pearson’s Correlation Coefficient the NIS Elements software version 4.03 (Nikon) was used. At least ten layers in 3D plane were scanned for each sample and for all samples a cropped image of a minimum of 12 cells was used to determine a total of 5 co-localization coefficients. The median value for all layers/cells of a given image was calculated and used to plot the Pearson’s Correlation Coefficient. Experiments were performed at least three times with three different healthy donors each time

### Western blotting

### Cytoplasmic and nuclear protein fractions of resting and activated T cells were separated using the NE-PER Extraction Kit (78833; ThermoFisher, Waltham, MA) following the manufacturer’s provided protocol. Twenty ng total protein per well was separated using 4 - 12% Tris Glycine gels (XP04120BOX ; ThermoFisher,) and proteins then transferred using iBlot nitrocellulose transfer stacks (IB301001; ThermoFisher) and iBlot transfer equipment (ThermoFisher) for 6 mins. Following blocking with fat-free milk (Marvel) for 2 hours at room temperature, the membranes were incubated over night with appropriate primary and/or secondary antibodies at the manufacturer’s suggested concentration/dilutions. Proteins were then visualized using the ChemiDoc Imaging System (BioRad, Watford, UK) in combination with Image Lab Software (BioRad, Hercules, CA). The Image Lab Software was also used to measure the intensity of the obtained bands via densitometric analysis.

**Supplementary Figure 1: AEP activity is required for normal Th1 induction in human and mouse CD4^+^ T cells.**

**Supplementary Figure 1. AEP is required for normal IFN-γ production in human and mouse CD4^+^ T cells. (A)** Cartoon representing the CD46 and IL-2-driven human Th1 cell life cycle. Left panel: autocrine activation of CD46 during T cell receptor (TCR) activation induces strong IFN-γ secretion and Th1 lineage induction in human CD4^+^ T cells as well as the assembly of IL-2 receptor (IL-2R) in the cell surface. Middle and right panels; during the expansion of a successful Th1 response, environmental IL-2 increases and CD46 and IL-2R signalling then drives the co-production of IL-10 in Th1 cells (through a yet undefined signalling pathway) and finally the switch into ‘self-regulatory’ IL-10 single positive contracting Th1 cells. **(B)** AEP inhibition reduces Th1 induction and IL-10 switching in human CD4^+^ T cells. Purified human CD4^+^ T cells were activated with the depicted antibody combinations in the presence or absence of 50 μM of a specific AEP inhibitor cytokine secretion measured at 36 hrs post activation using the CBA assay. Shown are the summarized data derived from *n*=5 healthy donors using a different donor each time. (**C**) AEP inhibition does not impact cell viability during CD4^+^ T cell activation. T cells were activated as under ‘B’ but with increasing amounts of an AEP inhibitor and cell viability measured 36 hrs post activation. (**D**) AEP is also required for normal IFN-γ secretion in mice. Purified naïve CD4^+^ T cells isolated from either wild type (WT) or AEP-deficient (*Lgmn*­^–/–^) mice (*n*=5) were activated for 6 days under Th1, Th2 or Th17 skewing conditions and the amounts of IFN-γ (Th1), IL-4 (Th2), and IL-17 (Th17) secreted into the cell culture media assessed. Error bar graphs represent mean ± SEM. **P* <0.05, ***P* <0.01, ****P* <0.001; ns, not significant.

**Supplementary Figure 2: AEP activity drives cathepsin L-mediated C3 activation in human CD4^+^ T cells.**

**Supplementary Figure 2. AEP activity drives cathepsin L-mediated C3 activation in human CD4^+^ T cells. (A)** Schematic representing the intracellular cathepsin L (CTSL)-mediated C3 activation ‘pathway’. In resting CD4^+^ T cells (left panel), CTSL cleaves complement C3 intracellularly tonicaly into bioactive C3a and C3b. C3a engages with the lysosome-located C3aR which in turn activates the mammalian target of rapamycin (mTOR) in a process that sustains T cell homeostasis. Right panel, upon T cell receptor (TCR) stimulation the ‘CTSL-C3a-C3aR’ system rapidly translocates to the cell surface where C3a and C3b engage their respective receptors, C3aR and CD46, respectively. Together these receptors induce signals to metabolically reprogram the cell required for productive Th1 responses manifested by IFN-γ secretion. **(B)** Cartoon depicting AEP-mediated CTSL maturation. CTSL is synthesized within T cells in a preproCTSL form which is cleaved to proCTSL and further processed to single-chain CTSL. AEP cleaves the single-chain CSTL to the double-chain CTSL form. (**C**) AEP inhibition strongly reduces CTSL-driven intracellular C3a generation. Human CD4^+^ T cells were left non-activated (NA) or activated with the depicted antibody combinations in the presence or absence of 50 mM AEP inhibitor and C3a generation assessed 36 hrs post activation by FACS. Shown is one data set derived from a representative donor. (**D**) AEP inhibition does not affect MHC II upregulation in activated human CD4^+^ T cells. Purified T cells were activated as depicted and MHC II expression measured by FACS analysis 36 hrs post activation. (**Di**) shows a representative FACS plot from one experiment, whilst (**Dii**) shows the statistical analysis performed using three distinct experiments (*n*=3). (**E**). AEP deficiency does not affect C3a generation in mouse CD4^+^ T cells. CD4^+^ T cells were isolated from either wild type (WT) or AEP-deficient (*Lgmn^–/–^*) mice and left non-activated (NA) or activated with antibodies to CD3 and CD28. Intracellular C3a presence was measured by FACS analysis 48 hrs post activation. (**Ei)** depicts the representative FACS plot of one WT and *Lgmn^–/–^* mouse and (**Eii**) shows the statistical analysis using three animals from each group. (**F**) AEP activity induces nuclear translocation of CTSL in T cells. Purified human CD4^+^ T cells were CD3+CD46 activated for 36 hrs and nuclear translocation monitored via (**Fi**) confocal microscopy in combination with (**Fii**) with a Pearson’s correlation coefficient analysis. At least ten layers in 3D plane were scanned for each sample and for all samples a cropped image of a minimum of 12 cells was used to determine a total of 5 co-localization coefficients. ***P* <0.01; ns, not significant.

**References:**

Liszewski, M.K., Kolev, M., Le Friec, G., Leung, M., Bertram, P.G., Fara, A.F., Subias, M., Pickering, M.C., Drouet, C., Meri, S.*, et al.* (2013). Intracellular complement activation sustains T cell homeostasis and mediates effector differentiation. Immunity *39*, 1143-1157.

Loak, K., Li, D.N., Manoury, B., Billson, J., Morton, F., Hewitt, E., and Watts, C. (2003). Novel cell-permeable acyloxymethylketone inhibitors of asparaginyl endopeptidase. Biol Chem *384*, 1239-1246.

Wang, G., Liszewski, M.K., Chan, A.C., and Atkinson, J.P. (2000). Membrane cofactor protein (MCP; CD46): isoform-specific tyrosine phosphorylation. J Immunol *164*, 1839-1846.
